# Modeling macrophage-T cell interactions in the breast cancer immune microenvironment: from spatial omics to functional validation

**DOI:** 10.3389/fimmu.2026.1870393

**Published:** 2026-07-14

**Authors:** Zhe Tian, Lijun He, Xiaojing Zhang

**Affiliations:** 1 Department of Breast Surgery, The Affiliated Hospital of Beihua University, Jilin, Jilin, China; 2 Department of Ultrasound, The Second Affiliated Hospital of Army Medical University, Chongqing, China; 3 Department of Breast Surgery, Jilin People’s Hospital, Jilin, Jilin, China

**Keywords:** breast cancer, functional validation, macrophage-T cell interactions, precision immunopharmacology, spatial omics, T cells, tumor microenvironment, tumor-associated macrophages

## Abstract

Breast cancer immunity depends on more than the number of immune cells in a tumor. It is also shaped by where those cells sit, which neighbors they contact, and what functional states they adopt locally. Tumor-associated macrophages (TAMs) and T cells are a key pairing in this setting. Depending on tissue context, their crosstalk may support cytotoxic immunity, reinforce immune exclusion, promote T-cell exhaustion, or weaken therapeutic response. Spatial technologies now allow these states to be examined in intact tumor sections rather than inferred from dissociated or bulk samples. Antibody-based imaging approaches, including imaging mass cytometry, MIBI, and CODEX, together with high-plex transcriptomic platforms such as MERFISH, Xenium, CosMx, Visium, GeoMx, and related methods, have revealed inflamed, excluded, myeloid-rich, stromal-barrier, and tertiary lymphoid structure-associated niches in breast cancer. However, spatial maps alone cannot establish mechanism. Cells that lie close together may not necessarily interact, and computational tools, including ligand-receptor scoring, graph-based neighborhood modeling, and spatial biomarker prediction, can only prioritize candidate macrophage-T cell programs. Functional validation remains essential. In this mini review, we discuss how spatial omics, computational modeling, organoid and explant cultures, microfluidic models, perturbation assays, and therapeutic testing can be linked to study macrophage-T cell crosstalk. We highlight a practical workflow in which spatial maps generate hypotheses, experimental systems test causality, and post-treatment profiling determines whether candidate interactions are remodeled by therapy.

## 1 Introduction

Breast cancer is not a single immunologic entity. Tumor-intrinsic programs, stromal organization, and local immune architecture vary across molecular subtypes, and these differences shape both disease progression and treatment response (1, 2). Although modern systemic therapy has improved outcomes for selected patients, TNBC remains clinically and immunologically heterogeneous (3). Many TNBCs contain higher levels of tumor-infiltrating lymphocytes than hormone receptor-positive tumors, whereas immune activation in HER2-positive disease is more variable and depends on molecular context and prior treatment (4). For this reason, immune cell abundance alone is difficult to interpret without knowing where those cells are located and how they are organized within the tissue.

Among immune populations, TAMs and T cells are especially important because both are highly plastic. CD8+ T cells can mediate tumor control, but exhausted effector cells and regulatory T cells may instead reflect ineffective or suppressive immunity. TAMs also occupy a wide functional spectrum, ranging from inflammatory and antigen-presenting states to pro-angiogenic, matrix-remodeling, and immunosuppressive programs (5). Therefore, macrophage-T cell contact is not automatically beneficial or harmful. Its meaning depends on macrophage state, T-cell differentiation, stromal context, tumor subtype, and treatment exposure. A macrophage-rich niche located near exhausted T cells may suggest immune suppression, whereas macrophages with antigen-presentation and interferon-response features may contribute to productive antitumor immunity.

Spatial omics now makes it possible to examine these relationships directly in intact breast cancer tissue. By combining spatial transcriptomics, multiplex imaging, and related platforms (6), investigators can localize immune-inflamed, immune-excluded, myeloid-rich, stromal-barrier, and tertiary lymphoid structure-associated niches (7–10). Such maps help determine whether TAMs accumulate at tumor-stromal borders, colocalize with exhausted CD8+ T cells, enrich regulatory T-cell neighborhoods, or participate in antigen-presenting immune niches. Still, a spatial map is not a functional assay. Proximity and ligand-receptor co-expression should be read as hypotheses that require computational prioritization and experimental testing, including organoid-immune co-cultures, tumor slices, explant models, perturbation assays, and therapeutic response studies (11, 12). Earlier single-cell and spatial surveys have catalogued many breast cancer immune and stromal populations, and recent reviews have comprehensively mapped emerging spatial technologies and their clinical applications across cancers (13); the present review focuses more narrowly on how these maps can be tested functionally (14). As a narrative mini review, this article draws on a non-systematic search of PubMed and Web of Science for English-language articles published up to 2026, combining terms such as breast cancer, spatial omics, spatial transcriptomics, tumor-associated macrophages, T cells, tumor microenvironment, cell–cell communication, and functional validation, supplemented by manual screening of reference lists; representative and recent studies were prioritized rather than exhaustively catalogued.

## 2 Spatial omics defines macrophage–T cell neighborhoods in breast cancer

### 2.1 Mapping immune architecture across breast cancer subtypes

Spatial omics has moved breast cancer immunology beyond simple immune-cell counting and toward a more anatomical view of the tumor microenvironment. Conventional immunohistochemistry, flow cytometry, and bulk transcriptomics showed that immune infiltration differs by subtype, with many TNBCs showing higher TIL levels and HER2-positive tumors displaying more variable immune activation (4, 15). However, these methods say little about where T cells are positioned: whether they enter malignant epithelial regions, remain confined to stroma, cluster around antigen-presenting cells, or are kept away from tumor nests by fibroblasts, vessels, extracellular matrix, or myeloid-rich barriers. Spatial transcriptomics and multiplex imaging help address this limitation by measuring immune phenotypes while preserving tissue architecture (16).

Across breast cancer subtypes, spatial studies have repeatedly described inflamed, excluded, desert, myeloid-rich, stromal-barrier, and tertiary lymphoid structure-associated patterns (7, 8). In inflamed tumors, CD8+ T cells, antigen-presenting cells, and inflammatory chemokines are found close to malignant epithelial cells. In excluded tumors, T cells may be present in substantial numbers but remain largely restricted to stromal regions, suggesting that fibroblast networks, abnormal vasculature, extracellular matrix, or suppressive myeloid cells limit direct engagement with tumor cells. Myeloid-rich niches often contain TAMs expressing scavenger receptors, angiogenic programs, matrix-remodeling molecules, and checkpoint ligands. By contrast, TLS-associated regions may bring T cells, B cells, dendritic cells, and high endothelial venule-like structures into closer spatial coordination.

TAMs are particularly informative in these ecosystems because they are distributed across several tissue compartments rather than occupying a single immune zone. Some macrophages localize near tumor nests and express antigen-presentation or interferon-response genes, whereas others accumulate in hypoxic, necrotic, perivascular, or stromal regions and show programs related to immunosuppression, angiogenesis, tissue repair, or matrix remodeling (17, 18). Their relationship with T cells is therefore context-dependent. A macrophage-enriched stromal rim may accompany T-cell exclusion, whereas macrophages in inflamed neighborhoods may support antigen presentation or local cytokine exchange. This is why spatial omics has encouraged a shift from a cell-counting model to an architectural model, in which neighborhood composition and tissue context help define immune function (19, 20). Platform choice also matters, since imaging-based spatial transcriptomic assays differ in tissue compatibility, segmentation quality, gene panels, throughput, and practical workflow (6, 21).

These platforms are not interchangeable, and platform choice should be driven by the biological question. For example, antibody-based imaging methods such as imaging mass cytometry (IMC), multiplexed ion beam imaging (MIBI), and CODEX are useful when the aim is to define macrophage and T-cell phenotypes, functional protein states, or direct cell-cell contacts at high spatial resolution. Their main limitation is that they measure a restricted set of markers and are less suited to broad discovery. By contrast, imaging-based transcriptomic methods such as MERFISH, Xenium, and CosMx can localize hundreds to thousands of transcripts at single-cell or subcellular resolution, making them useful for mapping fine neighborhoods and candidate ligand-receptor relationships, although panel design, segmentation quality, and optical crowding can affect interpretation. Visium, Visium HD, and GeoMx provide broader transcriptomic or region-level information and are valuable for discovery and tissue-context analysis, but they sacrifice some single-cell precision. Recent benchmark studies using matched FFPE tumor sections, including breast cancer samples, have compared these trade-offs in sensitivity, specificity, cell-type assignment, and panel effects across major commercial platforms (6, 21, 22). In many macrophage-T cell studies, the strongest design is therefore to combine protein- and RNA-based platforms on serial sections rather than relying on a single assay.

### 2.2 Spatial proximity and ligand–receptor inference of macrophage–T cell crosstalk

Macrophage-T cell crosstalk cannot be inferred simply from the presence of both populations in the same specimen. A more useful question is whether macrophages and T cells are positioned in a way that makes interaction plausible, and whether their local states suggest antigen presentation, cytokine exchange, checkpoint regulation, metabolic suppression, or stromal restriction. Spatial omics can therefore help identify macrophage-T cell neighborhoods that deserve mechanistic follow-up rather than treating all spatial proximity as functional contact (9, 10). For example, macrophages located near exhausted CD8+ T cells may express PD-L1, galectin family members, IL-10, or TGF-β-associated programs, whereas macrophages close to proliferating cytotoxic T cells may show interferon-response genes, antigen-presentation markers, or CXCL9/CXCL10 expression.

Several signaling axes are especially relevant to interpreting these neighborhoods, but they should not be viewed as equivalent. CCL2-CCR2 primarily supports recruitment of circulating monocytes into tumors, whereas CSF1-CSF1R is more closely linked to macrophage survival, differentiation, and maintenance. Together, these pathways shape TAM abundance and can indirectly influence whether T cells enter tumor nests or remain excluded. In inflamed regions, CXCL9/10-CXCR3 signaling may favor effector T-cell recruitment, whereas CXCL12-rich stromal areas may contribute to immune exclusion. Checkpoint interactions such as PD-L1-PD-1 and CD80/CD86-CTLA4 can limit T-cell activation. Other pathways, including SPP1-CD44/integrin signaling, TGF-β signaling, IL-10 signaling, and the CD39-CD73-A2A receptor axis, may connect macrophages with matrix remodeling, T-cell exhaustion, adenosine-mediated suppression, or regulatory T-cell enrichment (23, 24).

**Figure 1:**
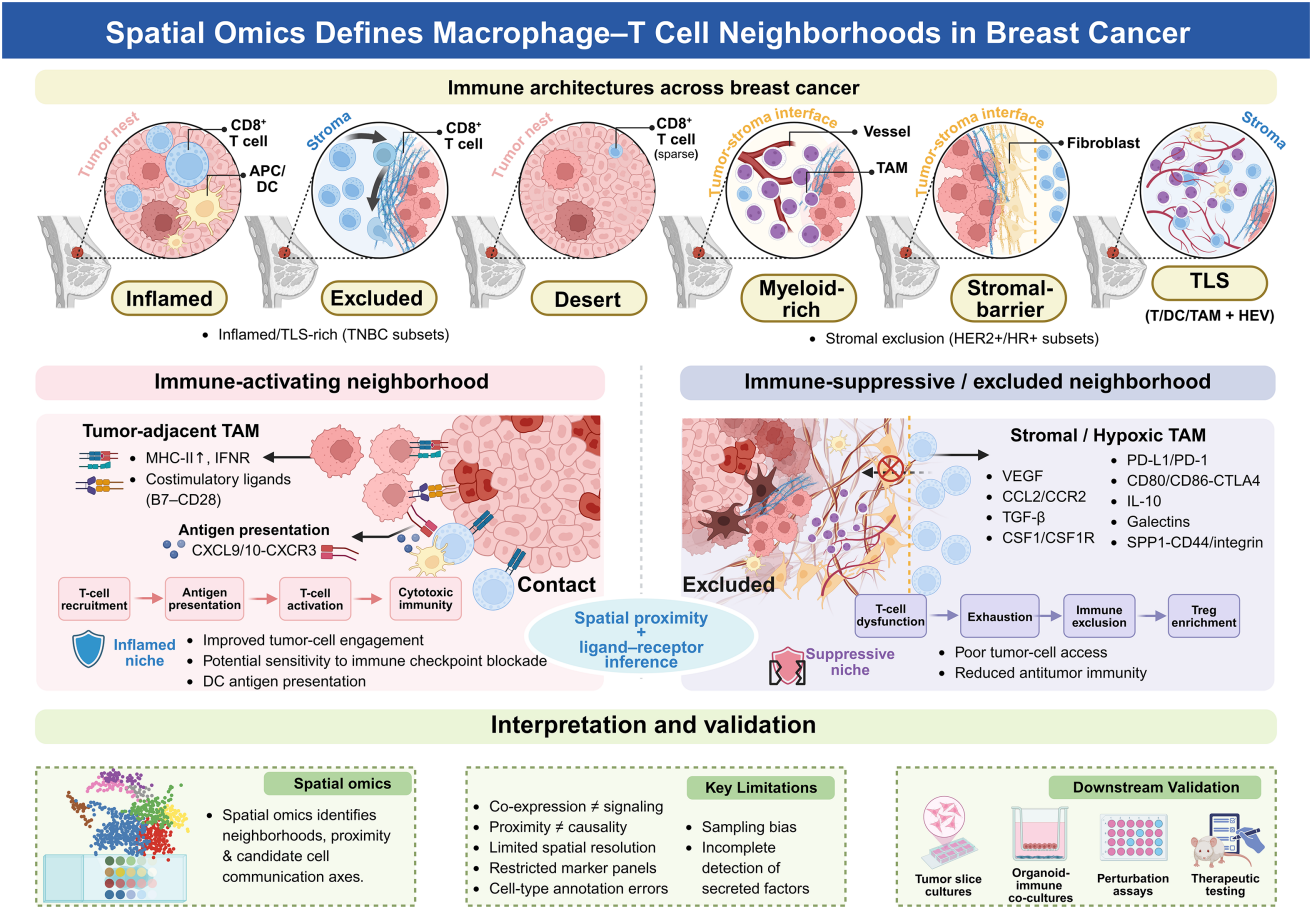
Spatial omics defines macrophage–T Cell neighborhoods in breast cancer (Image 1 was prepared using BioRender solely for schematic illustration and not for generating scientific data or text, under agreement number DY29TGQ5HX).

These inferences remain hypothesis-generating. Co-expression does not prove signaling, and proximity alone cannot distinguish active communication from passive coexistence. The technical limitations are also substantial: some spatial transcriptomic platforms do not provide true single-cell resolution, multiplex imaging panels are constrained by marker selection, and inferred networks can be distorted by annotation errors, sampling bias, or poor detection of secreted proteins and metabolites (25). For this reason, predicted macrophage-T cell interactions should be treated as ranked candidates for validation. The strongest studies will connect spatial prediction with perturbation experiments and functional readouts, such as T-cell killing, cytokine production, antigen presentation, checkpoint blockade response, or macrophage state change (**Figure 1**).

## 3 Computational modeling of macrophage–T cell ecosystems

Computational modeling provides a way to move from spatial description to testable biological priorities. Spatial omics can define cell identity, marker expression, transcriptional state, and tissue coordinates, but the key issue is whether a recurrent pattern reflects meaningful organization rather than random colocalization. Neighborhood analysis can help address this by identifying repeated structures, such as myeloid-rich stromal barriers, CD8+ T cell-tumor interfaces, TLS-associated regions, or myeloid-Treg niches. Tools such as Squidpy are useful for spatial statistics and neighborhood enrichment, whereas Tangram can help project single-cell information onto spatial measurements (26, 27). These analyses provide the first layer of prioritization, but they are most informative when followed by graph-based or communication models that ask which interactions may be driving a given niche or response-associated pattern (28, 29). Predictive modeling can then test whether these spatial features carry information about immunotherapy response (30).

Because these models are designed for different questions, they should not be used interchangeably. LIANA+ is helpful for comparing and consolidating cell-cell communication scores, including spatially informed analyses, whereas COMMOT models spatial communication using optimal transport under diffusion and competition constraints (31). MISTy is useful when the question is how intracellular, juxtacrine, and paracrine factors contribute to gene expression, while NCEM focuses on how a cell’s neighborhood shapes its transcriptional state (28, 32). In practice, these tools should not be treated as black boxes that simply produce interaction lists. Their value lies in whether they help make a concrete decision: which macrophage program should be perturbed, which macrophage-T cell neighborhood should be validated experimentally, or which spatial signature is worth testing as a response biomarker.

Beyond these communication- and niche-level tools, two further classes of methods are increasingly relevant. First, spatial deep-learning approaches use graph neural networks and related architectures to learn tissue organization directly from spatial data. Graph-attention and contrastive-learning models such as STAGATE and GraphST identify spatial domains and denoise expression while preserving tissue structure, and scalable frameworks such as CellCharter identify and compare cellular niches across many samples and platforms, including the kind of macrophage- and tumor-microenvironment-enriched niches relevant to breast cancer (33–35). These methods are well suited to defining reproducible macrophage-T cell neighborhoods at scale, although their outputs require biological interpretation and experimental confirmation. Second, agent-based and mechanistic models complement these descriptive approaches by simulating how individual cells move, signal, and respond to their microenvironment over time. Frameworks such as PhysiCell allow explicit simulation of cell-cell interactions, chemokine gradients, hypoxia, and treatment perturbations in a virtual tumor microenvironment, providing a way to test whether a hypothesized macrophage-T cell mechanism can reproduce observed spatial patterns and to explore candidate interventions in silico before experimental validation (36). Used together, data-driven spatial deep learning and mechanistic agent-based modeling can convert static spatial maps into dynamic, testable hypotheses about macrophage-T cell regulation.

For precision immunopharmacology, computational analysis is most useful when it goes beyond describing spatial patterns and produces predictions that can be tested. In TNBC, imaging mass cytometry data from neoadjuvant immune-checkpoint-blockade cohorts have shown that selected cell phenotypes and spatial cell-cell interactions can help predict treatment response (30). These findings suggest that neighborhood composition, marker co-expression, and contact frequency may serve as candidate spatial biomarkers rather than merely descriptive features. Newer frameworks have begun to formalize this step. For example, mosna extracts preferential cell-cell interactions and neighborhood features from spatial proteomic and transcriptomic data, then uses these spatial network features to train machine-learning models for predicting immunotherapy response and survival (37). The same principle can guide functional work: if a model links a macrophage-T cell neighborhood to nonresponse, the next step should be to test whether disrupting the suppressive signal or restoring productive T-cell activity changes the phenotype (**Table 1**).

**Table 1:** Computational methods for modeling macrophage-T cell interactions in spatial breast cancer datasets.

Method axis	Representative approaches	Question addressed in macrophage-T cell studies	Typical output and main caution
Spatial structure analysis	Squidpy; neighborhood enrichment and spatial graph statistics (26)	Which macrophage and T-cell states are recurrently adjacent, excluded, or enriched within tumor, stromal, myeloid-rich, or TLS-associated regions?	Contact frequencies, neighborhood-enrichment scores, and spatial autocorrelation. These outputs identify candidate niches but remain correlative.
Cell-cell communication inference	LIANA+; COMMOT; ligand-receptor and spatial-transport models (29, 31)	Which ligand-receptor axes may connect TAM programs with exhausted, cytotoxic, regulatory, or excluded T-cell states?	Ranked signaling pairs, communication flows, and spatial signaling directionality. Results depend on ligand-receptor databases, expression thresholds, and spatial resolution.
Multi-view niche and neighborhood-effect modeling	MISTy; NCEM; multiview and graph-based neighborhood-effect models (28, 32)	How do intracellular features, direct neighbors, and broader paracrine tissue context explain local cell-state variation?	View-specific feature importance, neighborhood effects, and context-dependent gene programs. Interpretation requires careful separation of association from causal signaling.
Spatial domain and niche identification	STAGATE; GraphST; CellCharter and related graph/deep-learning frameworks (33–35)	Which spatial domains or cellular niches are reproducible across samples, platforms, or clinical groups?	Latent embeddings, spatial clusters, domains, and cellular niches. Model-derived groupings need biological annotation and experimental validation.
Predictive spatial modeling	IMC-derived spatial predictors; mosna spatial-network features (30, 37)	Can macrophage-T cell composition, contact patterns, or spatial network features predict response to immunotherapy or survival?	Candidate spatial biomarkers and response/survival classifiers. Independent validation and cohort-size awareness are essential.
Mechanistic and agent-based simulation	PhysiCell and related agent-based tumor-microenvironment models (36)	Can a parameterized model test whether hypothesized cell behaviors, gradients, or contact rules reproduce observed spatial patterns and explore candidate perturbations?	Dynamic in silico perturbation scenarios. Utility depends on biologically realistic parameters and validation against experimental systems.

## 4 Functional validation of macrophage–T cell interactions

### 4.1 Ex vivo and organoid models for validating spatial hypotheses

Spatial omics can point to macrophage-T cell neighborhoods of interest, but it cannot by itself show whether those neighborhoods actually regulate immunity. This is where functional models become necessary. Organoids, tumor spheroids, tumor slices, explants, microfluidic chips, and autologous co-cultures each preserve different parts of the tumor-immune system (11, 38). Organoids are useful for maintaining epithelial heterogeneity and testing drug responses, although standard organoid cultures often lose the endogenous immune and stromal compartments that are central to macrophage-T cell biology. Tumor slices and explants are closer to the native tissue setting because they retain macrophages, T cells, fibroblasts, endothelium, extracellular matrix, and local spatial relationships. Their main drawback is practical: the viable experimental window is short, and controlled perturbation is often more difficult than in reductionist culture systems (12, 39).

Autologous co-culture systems can be used to test whether macrophages derived from patient tumors, monocytes, or induced differentiation systems suppress or support T-cell activity in a tumor-specific context. For example, macrophages identified spatially as PD-L1-high, SPP1-high, TGF-β-associated, or antigen-presentation-enriched populations can be isolated, enriched, or modeled *in vitro* and then tested with autologous T cells and tumor cells. Functional readouts should include T-cell infiltration, proliferation, cytotoxicity, IFN-γ and TNF-α production, granzyme B and perforin expression, checkpoint induction, T-cell receptor activation, macrophage polarization, antigen-presentation capacity, tumor killing, and live-cell imaging of cell-cell contact (40, 41). These assays help distinguish macrophage states that promote T-cell exclusion from those that support T-cell priming or effector activity. Microfluidic and 3D matrix-based systems further allow investigators to model chemokine gradients, tissue stiffness, stromal barriers, hypoxia, and directional migration, which are difficult to capture in conventional two-dimensional cultures (42).

The practical challenge is to balance physiological relevance against experimental control. Preserved explants are close to the native tumor but are difficult to scale and manipulate. Simplified co-cultures are easier to perturb but may miss stromal, vascular, and metabolic cues. A useful validation strategy is therefore staged: first test a predicted pathway in a tractable co-culture, then examine it in organoid-immune or tumor-slice systems, and finally look for the same remodeling pattern in treated preclinical or clinical samples. As noted above, perturbation is what separates a plausible spatial association from a mechanistically actionable macrophage-T cell interaction (12).

### 4.2 Perturbation strategies and therapeutic testing

Functional validation is most informative when it is tied to therapeutic perturbation. Macrophage-T cell interactions can be targeted by depleting suppressive macrophages, blocking monocyte recruitment, reprogramming macrophage state, improving antigen presentation, relieving checkpoint inhibition, or modifying stromal and metabolic barriers (23). CSF1R inhibitors can reduce or alter macrophage populations, and CCL2-CCR2 blockade may limit recruitment of monocytes that give rise to suppressive TAMs (43). CD40 agonists can activate antigen-presenting myeloid cells, while PI3Kγ inhibition has been explored as a way to shift macrophages from suppressive toward inflammatory states (44). Other macrophage-directed approaches, including PI3Kγ blockade and antibody-based reprogramming strategies, further illustrate how macrophage state can be manipulated therapeutically (45, 46).

Therapeutic testing should avoid the assumption that all macrophages are harmful. Some macrophage states support antigen presentation, cytokine production, tissue remodeling needed for immune access, or response to therapy. The more useful distinction is between indiscriminate depletion and state-specific reprogramming. Spatial omics can help identify whether a tumor contains macrophages forming suppressive stromal barriers, macrophages adjacent to dysfunctional T cells, or macrophages embedded in inflamed antigen-presenting niches. These patterns imply different interventions: a myeloid-rich excluded tumor may need macrophage reprogramming or recruitment blockade, whereas an inflamed tumor with suppressive checkpoints may be better suited to checkpoint-based combinations (5).

Perturbation models also provide a way to test treatment combinations before moving to expensive clinical studies. Chemotherapy and radiotherapy can induce immunogenic cell death and alter macrophage antigen presentation; HER2-targeted therapy, PARP inhibitors, antibody-drug conjugates, and endocrine therapy may reshape immune context in subtype-specific ways. Adding macrophage-targeting agents or checkpoint blockade may improve T-cell responses, but the best combination is likely to depend on the baseline spatial immune ecosystem (5). Useful assays should therefore measure not only tumor-cell death, but also T-cell entry, cytokine recovery, exhaustion markers, macrophage conversion, and post-treatment changes in macrophage-T cell proximity (**Table 2**).

**Table 2:** Functional validation models and perturbation strategies for macrophage-T cell interactions in breast cancer.

Validation/perturbation approach	Primary purpose	Key functional readouts	Advantages and limitations	Representative reference(s)
Patient-derived tumor organoids	Preserve tumor epithelial heterogeneity and drug-response features; reconstitute macrophage-T cell interactions when immune and stromal components are added.	T-cell infiltration; tumor-cell killing; cytokine release; checkpoint induction; macrophage polarization.	Useful for drug testing and patient-specific modeling, but standard organoids often lack endogenous immune and stromal compartments.	(11, 38)
Tumor slice cultures and explant models	Validate spatial hypotheses while retaining native tissue architecture, including macrophages, T cells, fibroblasts, endothelial cells, extracellular matrix, and spatial relationships.	Live-cell contact; T-cell activation; antigen-presentation capacity; macrophage state changes; tumor-cell death.	High physiological relevance, but short experimental windows, limited scalability, and less precise perturbation control.	(12, 39)
Autologous immune co-culture systems	Test whether patient-derived or modeled macrophages suppress or support T-cell activity in a tumor-specific context.	T-cell proliferation; IFN-γ and TNF-α production; granzyme B/perforin expression; TCR activation; tumor killing.	Strong mechanistic control, but may omit stromal, vascular, metabolic, and spatial cues present in vivo.	(41)
Microfluidic chips and 3D matrix-based systems	Model chemokine gradients, tissue stiffness, stromal barriers, hypoxia, and directional immune-cell migration.	T-cell migration; infiltration depth; chemotactic response; spatial redistribution; cell-cell interaction dynamics.	Captures spatial constraints better than 2D culture, but can be technically complex and harder to standardize.	(42)
Macrophage-targeted perturbation	Determine whether macrophage states exert causal immune-regulatory effects by depletion, recruitment blockade, reprogramming, or enhanced antigen presentation.	Macrophage abundance and phenotype; T-cell functional recovery; reduced exclusion; restored cytokine production; tumor-cell killing.	Can distinguish macrophage depletion from reprogramming, but must avoid eliminating macrophage states that support antitumor immunity.	(43, 44)
Combination therapeutic testing	Evaluate macrophage-targeting agents with checkpoint blockade, chemotherapy, radiotherapy, HER2-targeted therapy, PARP inhibitors, antibody-drug conjugates, or endocrine therapy.	Increased T-cell infiltration; reduced exhaustion; enhanced cytotoxicity; inflammatory macrophage conversion; improved tumor control.	Supports rational immunotherapy combinations, but optimal regimens depend on the pre-existing spatial immune ecosystem.	(47)
Spatial profiling linked to treatment response	Connect candidate macrophage-T cell niches with response-associated spatial features and clinically relevant treatment outcomes.	Macrophage-T cell proximity; immune exclusion; cell phenotype abundance; spatial interaction features; response-associated tissue states.	Creates a spatial prediction-validation link, but requires well-annotated treatment cohorts and careful modeling to avoid overinterpreting association as causality.	(30)
Spatial CRISPR/pooled perturbation screens	Link genetic perturbation to spatially resolved changes in tumor immune composition and macrophage–T cell organization in situ.	Spatially resolved gRNA detection; perturbation-associated shifts in immune-cell neighborhoods; macrophage and T-cell localization; niche remodeling.	Connects gene function directly to tissue-scale immune architecture, but is currently limited mainly to preclinical models, library size, and technical complexity.	(48, 49)

(APC, antigen-presenting cell; TCR, T-cell receptor; IFN, interferon; TNF, tumor necrosis factor; PARP, poly(ADP-ribose) polymerase; HER2, human epidermal growth factor receptor 2.).

## 5 Discussion and future perspectives

The next stage of breast cancer immune profiling should focus less on cataloguing additional cell states and more on connecting spatial states to druggable mechanisms (47). Spatial omics has already shown that breast tumors contain inflamed, excluded, myeloid-rich, stromal-barrier, and TLS-associated niches. The unresolved question is which of these patterns can be changed by treatment and which are merely descriptive. Macrophage-T cell interactions are especially challenging because the same broad macrophage label can include antigen-presenting, inflammatory, tissue-repair, angiogenic, immunosuppressive, and metastasis-associated programs. A spatial signature is therefore clinically useful only if it predicts a functional dependency or a treatment-induced shift. Patient-derived organoid-immune co-cultures, tumor slices, microfluidic systems, spatial CRISPR screens (48, 49), and ex vivo pharmacologic testing can help distinguish predicted interactions that are causal from those that are incidental. At the clinical end of this workflow, checkpoint-chemotherapy trials in advanced TNBC show why spatial biomarkers need to be interpreted alongside treatment context and patient selection (50, 51). TLS biology is also relevant because organized lymphoid niches can shape antitumor immunity and immunotherapy responsiveness across cancers (52). In melanoma, TLS and B-cell-associated immune structures have been linked with improved immunotherapy response and survival (53, 54). Similar B-cell-enriched immune contexts have been associated with survival and immunotherapy response in sarcoma, supporting the broader use of TLS as a cross-tumor spatial immune concept (55). By linking spatial organization to perturbation response, breast cancer immunology can move toward treatment strategies that match macrophage-targeting, T-cell-directed, stromal-remodeling, and combination approaches to the immune architecture of an individual tumor (47).
